# The Core Human Microbiome: Does It Exist and How Can We Find It? A Critical Review of the Concept

**DOI:** 10.3390/nu14142872

**Published:** 2022-07-13

**Authors:** Itai Sharon, Narciso Martín Quijada, Edoardo Pasolli, Marco Fabbrini, Francesco Vitali, Valeria Agamennone, Andreas Dötsch, Evelyne Selberherr, José Horacio Grau, Martin Meixner, Karsten Liere, Danilo Ercolini, Carlotta de Filippo, Giovanna Caderni, Patrizia Brigidi, Silvia Turroni

**Affiliations:** 1 Migal-Galilee Research Institute, P.O. Box 831, Kiryat Shmona 11016, Israel; 2 Faculty of Sciences and Technology, Tel-Hai Academic College, Upper Galilee 1220800, Israel; 3 Unit of Food Microbiology, Institute of Food Safety, Food Technology and Veterinary Public Health, Department for Farm Animals and Veterinary Public Health, University of Veterinary Medicine Vienna, A-1210 Vienna, Austria; narciso.m.quijada@vetmeduni.ac.at (N.M.Q.); evelyne.selberherr@vetmeduni.ac.at (E.S.); 4 Austrian Competence Centre for Feed and Food Quality, Safety and Innovation, FFoQSI GmbH, A-3430 Tulln an der Donau, Austria; 5 Department of Agricultural Sciences, Division of Microbiology, University of Naples Federico II, 80055 Portici, Italy; edoardo.pasolli@unina.it (E.P.); ercolini@unina.it (D.E.); 6 Task Force on Microbiome Studies, University of Naples Federico II, 80055 Portici, Italy; 7 Unit of Microbiome Science and Biotechnology, Department of Pharmacy and Biotechnology, University of Bologna, 40126 Bologna, Italy; m.fabbrini@unibo.it (M.F.); silvia.turroni@unibo.it (S.T.); 8 Microbiomics Unit, Department of Medical and Surgical Sciences, University of Bologna, 40138 Bologna, Italy; patrizia.brigidi@unibo.it; 9 Institute of Agricultural Biology and Biotechnology (IBBA), National Research Council (CNR), Via Moruzzi 1, 56124 Pisa, Italy; francesco.vitali@ibba.cnr.it (F.V.); carlotta.defilippo@ibba.cnr.it (C.d.F.); 10 Microbiology and Systems Biology, Netherlands Organization for Applied Scientific Research (TNO), Utrechtseweg 48, 3704 HE Zeist, The Netherlands; valeria.agamennone@tno.nl; 11 Department of Physiology and Biochemistry of Nutrition, Max Rubner-Institut (MRI)-Federal Research Institute of Nutrition and Food, 76131 Karlsruhe, Germany; andreas.doetsch@mri.bund.de; 12 Amedes Genetics, Amedes Medizinische Dienstleistungen GmbH, 10117 Berlin, Germany; josehoracio.graujipoulou@amedes-group.com (J.H.G.); martin.meixner@amedes-group.com (M.M.); karsten.liere@amedes-group.com (K.L.); 13 Center for Species Survival, Smithsonian Conservation Biology Institute, Washington, DC 20008, USA; 14 NEUROFARBA Department, Pharmacology and Toxicology Section, University of Florence, Viale Pieraccini 6, 50139 Florence, Italy; giovanna.caderni@unifi.it

**Keywords:** core microbiome, healthy microbiome, immune system, diet, prokaryotes, eukaryotes, virome, gut, NGS sequencing, omics

## Abstract

The core microbiome, which refers to a set of consistent microbial features across populations, is of major interest in microbiome research and has been addressed by numerous studies. Understanding the core microbiome can help identify elements that lead to dysbiosis, and lead to treatments for microbiome-related health states. However, defining the core microbiome is a complex task at several levels. In this review, we consider the current state of core human microbiome research. We consider the knowledge that has been gained, the factors limiting our ability to achieve a reliable description of the core human microbiome, and the fields most likely to improve that ability. DNA sequencing technologies and the methods for analyzing metagenomics and amplicon data will most likely facilitate higher accuracy and resolution in describing the microbiome. However, more effort should be invested in characterizing the microbiome’s interactions with its human host, including the immune system and nutrition. Other components of this holobiontic system should also be emphasized, such as fungi, protists, lower eukaryotes, viruses, and phages. Most importantly, a collaborative effort of experts in microbiology, nutrition, immunology, medicine, systems biology, bioinformatics, and machine learning is probably required to identify the traits of the core human microbiome.

## 1. Introduction

The importance of the human microbiome to our health has been demonstrated in numerous studies in recent years. Our microbiome is involved in various processes in our body including nutrient uptake [1,2], defense against pathogens [3], and the development of the immune system in newborns [4]. Microbes that inhabit our gut metabolize carbohydrates, proteins, vitamins, and other nutrients [5]. The human microbiome has been linked to different health conditions such as inflammatory bowel diseases (IBD), in which changes in the gut microbiome were observed in IBD patients, as well as mutations in genes related to the interactions between the immune system and the microbiome [6]. Links between the gut microbiome and the brain (the gut–brain axis) have been reported for multiple conditions such as depression and autism, and animal-based experiments show that fecal transplant can affect neuropsychiatric conditions [7]. While it is still not clear whether the human microbiome is causally related to these and other health conditions, it is clear that our microbiome is central to our health. The human microbiome has been the subject of significant works by thousands of researchers worldwide and multiple large national and international projects such as the Human Microbiome Project (HMP) [8,9] and MetaHIT [10].

Identifying a common set of stable components—a core microbiome—has been a major goal in human microbiome research for decades [9]. The main contribution of the core microbiome may be in the functions it provides to the human host. Further, dysbiosis may be caused by impaired functionality of the core microbiome in the holobiontic system, which leads to disease states [11]. From the ecological perspective, members of the core microbiome may not interact directly with the human host but contribute to the stability of the (core) microbiome. This can occur through metabolite processing, nutrient synthesis or in other ways. The human microbiome is highly individualized, with similarities observed between close family members [12,13]. At the individual level, the microbiome changes over time but certain species and strains may occupy the body for years [14,15]. Strains are transferred from mothers to their children during labor and later through breastfeeding [16]. Characterization of the core microbiome has been attempted repeatedly [12,13,17,18,19,20]. Nonetheless, it remains unclear whether patterns relating to this concept can be observed at the community composition, function, ecology, or some other level. Consequently, the core microbiome remains an elusive concept that is difficult to evaluate and investigate in microbiome research. In fact, it is difficult to assess whether the core microbiome even exists.

In this review, we consider the state of research on the core human microbiome. Other reviews on the topic focused on the concepts and terminology that should be used to define the core human microbiome in terms of common sets of taxa or functions [9,19,21,22,23,24]. Actual attempts to define the core and healthy human microbiome are based on genes and community compositions derived from 16S and metagenomics data [12,13,17,20]. In contrast, we consider the different information sources that can help achieve an evidence-based model of the core human microbiome. We assess the technological, methodological, and conceptual gaps that should be addressed to consider these levels of information. Specifically, we consider the present state and limitations of omics technologies and bioinformatics methods, and estimate the microbial and host dimensions that cannot currently be evaluated or that do not receive sufficient attention, and that may contribute to core human microbiome research.

## 2. Defining the Core Microbiome

The concept of the core human microbiome describes the stable components of the microbiome, both over time and between individuals. This contrasts with the transient aspects of the microbiome, which vary according to fluctuations in environmental and individual conditions such as diet, host genetics, and other, mainly exogenous factors. The stability of a microbiome is considered in its capacity to resist changes caused by a perturbation (resistance) and in the capacity to return to equilibrium after a perturbation (resilience) [23,24]. Stability is maintained at the level of the individual over a relatively short-term and over the long-term evolutionary scale at the population/species level, including changes in the microbiome during the evolution of the host species [18]. Early attempts to define the core microbiome focused on the set of genes present in the vast majority of humans [9]. However, the term has evolved as studies have offered different perspectives on the nature of the microbiome [25], including community composition and ecological parameters. For host–microbiome systems, the core microbiome is considered in terms of important microbes and functions for the host [26]. Any definition of a core microbiome should account for the diverse habitats for microbial life in the human body, which harbor their distinct microbiota [27]. Table 1 summarizes the main approaches for defining the core human microbiome. We focus on the two most commonly used approaches, community composition, and functional profile.

A **community-based** definition of the core microbiome searches for taxa consistently found in the studied host population. Accordingly, a core microbiome consists of members of the community that are common to multiple communities across different hosts. Other than the mere existence or nonexistence of specific taxa, features that can be considered include abundance, the occurrence of related taxa (as a proxy for functional redundancy), periodic re-occurrence (persistence), and correlation and anti-correlation of taxa (connectivity) [19]. A community-based definition of a core human microbiome assumes that its members contribute to the host’s health, either directly, through functions beneficial to the host, or indirectly, through contribution to community stability. Of particular interest are keystone species that play crucial roles in the ecological structure of their microbial communities and that are therefore essential for maintaining the organization and functions of these microbiomes [33]. Because of the disproportionate effect of keystone species on microbial ecosystems, the loss of these species can dramatically change the ecological niches of a microbiome. This may lead to dysbiosis and, for host-microbe systems, affect the capacity to perform functions that are important for the host; the clinical implications may be significant [34].

A **function-based** description of the core microbiome focuses on the set of functions that are consistent across populations at the level of genes or pathways. This approach is potentially more realistic than a community-based approach because it considers that multiple species can sometimes fill the same niche and provide the same functions. Functional redundancy ensures that certain functions in a microbiome may be performed by different species [35]. For example, in the human gut, multiple microbial species are responsible for the degradation of complex carbohydrates and the production of specific metabolites, ensuring the stability of the microbiome [36]. In this case, a specific functional capacity rather than the presence of certain taxa could be the crucial element that defines the core. 

**Figure 1:**
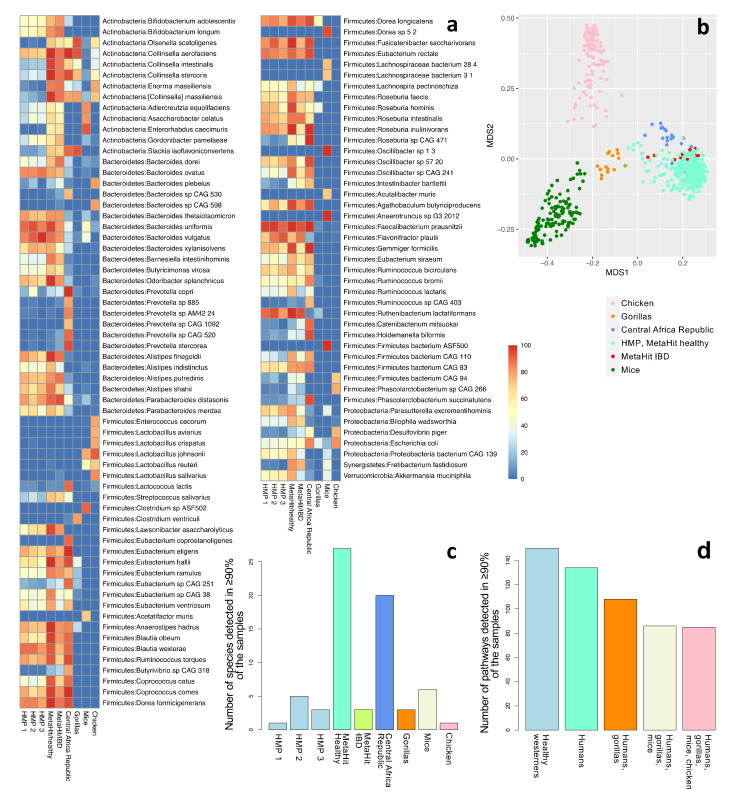
Community profiling of nine cohorts: HMP phases 1 (*n* = 138), 2 (*n* = 91), and 3 (*n* = 42); healthy individuals from Denmark (*n* = 64); individuals with IBD from Spain (*n* = 16); hunter-gatherers and traditional agriculturalists (*n* = 19); gorillas (*n* = 15); mice (*n* = 141); and chickens (*n* = 121). (**a**) The fraction of samples that contain each species, for species detected in at least 70% of the samples in at least one cohort (a total of 107 species). Refer to Supplementary Table S3 for a complete list of all the detected species in all cohorts. (**b**) The first two components of an unweighted UniFrac-based MDS analysis considering all samples. (**c**) The number of species detected in at least 90% of the samples of each cohort. Only one species (*F. prausnitzii*) was detected in 90% or more of the samples across all healthy human Western (*n* = 4) and all human (*n* = 6) datasets. (**d**) The number of pathways detected in >90% of the samples in all healthy Western human datasets (*n* = 4); all human datasets (*n* = 6); human and gorilla datasets (*n* = 7); human, gorilla, and mouse datasets (*n* = 8); and all datasets, including chickens (*n* = 9). See also Supplementary Tables S4 and S5.

To illustrate the challenges in defining the core microbiome, we consider the community and functional profiling of a relatively small dataset composed of 370 human and 277 animal samples from nine cohorts of fecal samples. These include healthy Westerners from the US (HMP phases 1, 2, and 3) [9,37] and Denmark (MetaHIT), individuals with IBD from Spain (MetaHIT) [28], and a group of hunter-gatherers and traditional Bantu agriculturalists from the Central Africa Republic [38]. As controls, we consider fecal samples from gorillas [38], mice [39], and chickens [40]. Community composition was evaluated using MetaPhlan3, and functional profiles were generated using HUMAnN3 [41] (see Supplementary Information for more details). Overall, 107 species were detected in at least 70% of at least one cohort (Figure 1a). As expected, abundant species profiles were more similar between the human cohorts than between humans and mice, gorillas, and chicken. An ordination plot highlights remarkable clustering of microbial composition in the function of the host species; Westernized and non-Westernized human populations are distinct. Moreover, human microbiomes tend to be more similar to the microbiomes of gorillas than to those of mice and chickens (Figure 1b). However, only a few species were typically identified in more than 90% of the samples within each cohort, and many of the highly prevalent species in one cohort were not as prevalent in other human cohorts (Figure 1c). Only one species, *Faecalibacterium prausnitzii*, was detected in at least 90% of the samples in all six human cohorts. Even this result may be questioned, because MetaPhlAn3 falsely identifies multiple *Faecalibacterium* species as *F. prausnitzii* and may therefore lead to false positive detection of this species [42]. These results show the inadequacy of a simplistic species-based approach for describing a community-based core microbiome. Considering the function-based approach, we identified 134 pathways shared among >90% of the samples in all the human cohorts, of which 85 were also shared with gorillas, mice, and chickens (Figure 1d and Supplementary Figure S1). Several studies identified a number of functions that are shared among many individuals as the possible core microbiome [9,12,13]. However, as many of these functions are also shared with other organisms, defining human-specific core microbial functions is difficult. Various genes that encode widespread functions may be unrelated to the interactions between the microbiome and its human host or to community stability, for example, housekeeping genes.

The difficulties in identifying a set of universal taxa or human-specific functions that can be regarded as a core human microbiome were also noted in previous studies on the topic [25]. These difficulties may arise because of the algorithms and databases available, or because of our focus on the genomic level instead of other, more relevant levels. It is also possible that our definitions of the core microbiome do not capture its essence. Next, we consider each of these aspects and try to evaluate their importance for the study of the core human microbiome. Table 2 summarizes the issues considered in this review. The rest of the paper is organized as follows. In Section 3, we consider the sources of data that are currently available; in Section 4, we describe the state of 16S and metagenomics bioinformatics and data sharing; in Section 5, we go beyond prokaryotic meta-omics to evaluate other fields that may contribute to our understanding of the core human microbiome; finally, in Section 6 and Section 7 we discuss the path forward in core human microbiome research.

## 3. The Data: Mostly Genomics, Western-Focused, and Fecal-Based

### 3.1. Technology: Mostly Genomics

Since the development of culturing-free techniques for studying microbial communities, microbiome research has shifted chiefly to relying on amplicon-based community surveys and metagenomics. As of March 2022, the European Nucleotide Archive (ENA) contains more than 610K human microbiome samples, of which more than 85% are estimated to be 16S rRNA amplicon samples (see Supplementary Information for for the analysis information). These, together with numerous samples available through other platforms, provide an amount of data that could potentially have been sufficient for evaluating the core microbiome of humans. The vast majority of available data was achieved using **amplicon-based community surveys**, which aim—through PCR and a specific set of primers—at a conserved region in the genomes of the target population. In microbiome surveys, one or more variable regions of the 16S rRNA gene are usually used to target prokaryotes. **Metagenomics** is based on untargeted (“shotgun”) sequencing of potentially all microbial genomes present in a sample [43]. Metagenomics data can be used to assess community composition and functional potential, and even for the reconstruction in silico of the sequences from the original genomes that were present in the samples (also known as “metagenome-assembled genomes” or “MAGs”). Aspects of metagenomics and amplicon data analysis that limit our ability to define the core microbiome will be discussed later. Other omics approaches, such as metatranscriptomics, metaproteomics, and metabolomics, could have been used for characterizing the core human microbiome at levels that represent the actual functional activity of the microbiome. Examples of functions identified at the transcription level and not at the gene level are known [44]. Assuming that the core human microbiome provides specific functions to the human host, these functions, or the lack of them in dysbiosis and disease, may be manifested at the levels of transcription and translation, and not at the genomic level. Currently, only a small amount of omics data beyond the genomics level is available. For example, we were able to identify less than 1500 human microbiome-related RNA-seq samples in ENA, the majority of which have a shallow sequencing depth of <100 Mbp (see Supplementary Information for for the analysis information). To the best of our knowledge, no study to date addressed the core human microbiome topic at a level other than the genomic. Data from large-scale multi-omics projects such as ibdmdb [45] are expected to facilitate such studies.

### 3.2. Population: Western-Focused

The core human microbiome differs substantially between geographical locations and populations [46]. Differences are not restricted to the gut microbiome but are also observed in other body habitats such as saliva and the vagina. Defining the core human microbiome therefore requires sufficient data for different populations. However, According to a recent estimate, more than 70% of human microbiome data deposited in public databases have been collected from Westerners [47]. Large-scale national and international efforts to study the human microbiome have mostly focused on American and European populations. These include the Human Microbiome Project [8,27,48], the Dutch LifeLines [49], the Belgian Flemish Gut Flora Project [50], and the MetaHIT Project [10]. However, only a few studies evaluated the gut microbiome in rural communities in Western countries whose diet consists of more traditional products, including those obtained from household plots, yet still in a modern environment [51,52]. In addition, as mentioned above, several studies shed light on the peculiarities of the gut microbiome of non-Western populations, including hunter-gatherers and rural agriculturalists [53,54,55,56,57,58,59,60,61]. While their sample sizes were generally small, these studies consistently reported different composition and functionality of the intestinal microbiome between urbanized and rural populations, with diet, lifestyle, or more generally, the exposome (i.e., the totality of exposures that each individual faces in the course of life) [62] exerting a pivotal role [63,64,65]. Clearly, the human gut microbiome varies across geography and subsistence practices [66,67]. Therefore, more data for rural and non-Western populations are required to obtain a reliable description of the core human microbiome.

### 3.3. Sample Source: Mostly Fecal

The various segments of the gastrointestinal tract of mammals differ in anatomy and function; the microbiome composition of these segments also differs [68]. In addition to the differences between gut segments, within a single gut segment, microbial function and prevalence vary between luminal and mucosal bacteria [69,70,71,72]. Oxygen gradients have been found to correlate with radial partitioning of microbial phylotypes in the colon [72,73]. Still, most reports on the human gut microbiome rely on fecal samples to represent the gut microbiome [74]. Fecal material is easy to access and does not require invasive technology. However, fecal microbiota represents only the last part of the colon’s luminal fraction; microbiota from other segments may elucidate microbe–host interactions and disease progression in the gut [71]. 

A certain core microbiome may fit the conditions of each specific microenvironment (niche) of the gut, with varying significance to human health. For example, microbes associated with mucosal sites were shown to have more significant effects on immunity and health parameters compared to luminal and fecal microbes [75,76]. Compared to the fecal microbiota, mucosal microbiota also better differentiate individuals with IBD from controls [77]. Dysbiosis, which leads to the loss of keystone community members in the mucosa may be more significant to identify. However, the mucosal microbiome cannot be evaluated according to fecal samples, as differences between the mucosal and fecal microbiome are evident in the healthy state, in IBD, and in irritable bowel syndrome [77,78,79], as well as in cirrhosis and hepatic encephalopathy [80].

If a core human microbiome exists, we may be able to characterize it in body parts that have fewer microenvironments, or in body parts that are more easily accessible. Compared to the gut, other parts of the human body have been sampled less extensively (see Supplementary Table S1). On the upper end of the digestive tract, the oral cavity represents another environment, whose complex mixture of habitats comprises distinct microbiomes [17,81]. The mouth is much more accessible than the gut and was sampled at nine distinct sites in the HMP [27]. Moreover, specific databases of the human oral cavity exist, such as the extended Human Oral Microbiome Database. This database contains over 700 bacterial species isolated from the human mouth and aerodigestive tract, including the pharynx, nasal passages, sinuses, and esophagus, thus enabling more accurate sample-specific microbiome annotations. However, attempts to define a core human oral microbiome have focused on saliva [82,83,84,85], which also provides a restricted view of the overall diversity.

## 4. The Methods: Low-Resolution Amplicon Studies, Challenging Metagenomics Bioinformatics, and Difficult to Mine Public Databases

The analysis of microbial communities at the genomic level heavily relies on 16S rRNA gene surveys, which provide community composition information (“who is there?”), and metagenomics, which also provides functional information (“what can they do?”). Community analysis is performed in terms of ecological measures such as the alpha and beta diversity, lists of taxa and functions, and statistical methods for identifying features enriched in certain groups of samples. Genome reconstruction from metagenomics data helps expand our knowledge of species living in different environments and their potential metabolic and physiological features. Furthermore, 16S surveys and metagenomics are key techniques in defining the core human microbiome. However, both exhibit limitations that affect our ability to define the core human microbiome.

### 4.1. 16S rRNA Gene Surveys: Established Bioinformatics, and Low-Resolution and Partial Information

Amplicon-based studies are the most cost-effective option to investigate microbial populations, and several established computational pipelines for evaluating community composition are available [86,87,88]. Furthermore, the high sequencing depth achieved by the most popular Illumina platforms enables in-depth characterization of bacterial/archaeal communities present in an environment, including identification of low abundance microorganisms [89]. Additionally, 16S rRNA gene surveys are considered a semi-quantitative approach as, despite the intermediate PCR step during library preparation, the number of reads obtained for a given taxon is proportional to its abundance in the sample [90].

Several significant limitations of 16S rRNA gene surveys hinder their ability to evaluate the core human microbiome. First, 16S rRNA surveys only partially characterize the microbial community based on a set of selected primers. Eukaryotes and viruses are not represented, nor are lineages of archaea and bacteria that do not fit the primers used. Also, the taxonomic resolution of 16S rRNA gene surveys is low and does not exceed the genus level at best. This is due to the short length of the sequenced variable regions, typically at the level of genus at most [91], and precludes distinguishing between closely related species [92,93]. Strategies for improving resolution, including the use of multiple variable regions simultaneously [94], and the use of other marker genes [95], have been proposed but are not yet widely used. In addition, between one and 15 copies of the 16S rRNA gene (median = 5, according to the Ribosomal RNA Database, https://rrndb.umms.med.umich.edu/, accessed on 20 March 2022) are known to exist in a number of bacteria [96], and variability in the sequence of the copies within the same organism has been described [96]. This may lead to false evaluation of relative abundance and false identification of amplicon sequence variants (ASVs) or operational taxonomic units (OTUs) due to intra-organism variation in the gene’s sequence [97]. Finally, it is unclear how OTUs and ASVs can be linked to taxonomic levels that facilitate a common language such as strains and species.

These issues, in addition to the lack of relevant functional information, limit the contribution of 16S rRNA gene surveys to the study of the core human microbiome. High-level taxa that consistently appear in various populations can be identified using 16S surveys. However, the current methodology cannot evaluate their potential contribution to the core human microbiome, and the exact species and genes that contribute to the core microbiome.

### 4.2. Metagenomics: High-Resolution Taxonomic and Functional Data, Challenging Bioinformatics

While it is still not clear which taxonomic level (if any) should be considered for defining the core human microbiome, studying the microbiome at the species level is desired. Gene content of the microbiome is essential for applying a functional-based definition of the core microbiome. In theory, metagenomics facilitates characterizing the entire microbial community, including both community composition at the strain level and functional profiling, for both previously seen and unseen species [41,98]. Unlike 16S rRNA gene surveys, in metagenomics, the total DNA extracted from the sample is directly subjected to shotgun sequencing without a targeted PCR amplification step [99]. On the other hand, metagenomics is far more expensive than amplicon-sequencing surveys and is challenging to implement when the studied environment is rich with host DNA, as in mucosal-associated microbiome studies [100]. In addition, metagenomics data analysis requires significant computational resources, and knowledge of bioinformatics and programming is required for analyzing the data [101]. As a result, while the number of publicly available metagenomes is small compared to 16S rRNA gene surveys, analyzing these samples to evaluate the core microbiome is currently challenging. 

Both taxonomic composition and the functional profile of metagenomics samples need to be evaluated to identify the core human microbiome. Assembly-free algorithms that rely on read-mapping of the raw data against reference databases can potentially accurately describe the community, including its rare members, with relatively low computational resources. Multiple such methods and pipelines are available, including MetaPhlAn3 [41], Kraken and Bracken [102,103], Centrifuge [104], and mOTUs2 [105], for species-level community composition, and HUMAnN3 [41] for functional profiling. The accuracy and sensitivity of these methods rely heavily on the availability of comprehensive reference databases [106]. Half or more of the species in the human microbiome have been estimated to lack representatives in public databases [67]. Moreover, up to 40–70% of the predicted proteins do not have a known function even for well-studied environments such as the human body [107]. Therefore, to close these gaps, increased effort is required in microbial culturing, biochemical investigations, and genome recovery for uncharacterized species. Assembly-based approaches that rely on de novo assembly of the reads into contiguous contigs and scaffolds can be used. Assembly, followed by gene prediction and the *binning* of contigs and scaffolds into MAGs that represent community genomes of microbial community members, can enrich our knowledge of microbes that evade culturing [108,109]. Indeed, hundreds of thousands of MAGs were reconstructed from human-associated metagenomes in recent years [67,110,111], contributing to the characterization of previously unknown lineages in the tree of life. Once incorporated into public databases, these MAGs facilitate large-scale and accurate species-level profiling of the human-associated metagenomes needed to evaluate the core microbiome.

### 4.3. Public Microbiome Datasets Are Often Hardly Interoperable and Reusable

Regardless of the adopted theoretical definition of the core human microbiome, its large-scale practical definition will inevitably be subjected to extensive cross-study meta-analysis. In defining the core human microbiome, data from a large number of individuals from several studies should be integrated and analyzed. This requires metadata such as ethnicity, geography, and disease state, and information regarding sample processing. Effective data reuse has been widely explored in all fields of science, especially from the advent of “omics” techniques, and as inspired and guided by the 2016 publication of the FAIR data (findability, accessibility, interoperability, and reuse of data) principle [112]. Unfortunately, performing meta-analyses is still challenging due to biases related to sample processing and the lack of metadata.

Sample processing involves multiple steps, which can introduce biases, such as in sample collection and storage, DNA extraction protocols, PCR, the choice of variable regions and primers (in 16S rRNA gene surveys), sequencing technology, and the bioinformatics pipeline used. The biases can range from eliminating taxa to reporting skewed relative abundances [113,114,115,116]. Therefore, all the information relevant to sample processing should be deposited with the data, to account for these issues and to select datasets that are comparable (i.e., have undergone similar processing steps). Unfortunately, this information is currently primarily available in the publication of the data, but usually not as part of the metadata deposited with the data. 

***Making data public does not guarantee their effective reuse.*** Journals and funding agencies usually require the upload of raw sequences to public databases; this constitutes a *de facto* obligation for the authors to make the data publicly accessible. However, the availability of raw data does not in itself ensure effective data reuse, and metadata (i.e., the diversificated set of data and information describing the subjects of a study) are required, but are often missing or incomplete [117]. For successful data reuse, a rich set of metadata that is standardized using ontologies should always accompany raw data. The ENA [118] and NCBI’s Sequence Read Archive (SRA) [119] allow uploading a high volume of metadata as sample attributes, using well-defined checklists derived from templates and suggestions developed by international initiatives as the minimum information about any (x) sequence (MIxS) specification [120]. In addition, users are suggested to employ words derived from well-defined and standardized vocabularies/ontologies such as ENVO (Environment Ontology; [121]), CHEBI (Chemical Entities of Biological Interest; [122]), and DO (Disease Ontology; [123]), for the annotation of metadata of crucial samples. Examples of such data are the material feature that was the sample source (i.e., feces from the human gut environment), the reference to any chemicals used, and the pathology state. Still, the use of most of those metadata is not mandatory, and crucial information for effective meta-analysis, such as the host disease state and demographics, is often missing. We believe that adherence to a few guidelines considering metadata deposition and collection can greatly improve microbiome data reusability. However, given that core microbiome research can greatly benefit from meta-analyses of many datasets, the current state of public microbiome data poses obstacles.

## 5. Beyond Prokaryotic Omics: Interactions of the Core Human Microbiome with Its Host, and with Other Members of the Microbiome

### 5.1. Specific Effects of Diet on the Microbiome

Microbial communities comprise a multitude of species interrelated by the trade of metabolites, some acting as primary energy substrates whilst others (e.g., secondary metabolites, by-products, and wastes) as substrates for other members [124]. The stability of a colonizer is determined by a combination of biotic and abiotic factors; biotic factors are represented by interactions with other members of the community, and abiotic factors by the availability of nutrients or the presence of harmful compounds in the environment. Diet is considered the main determinant of the compositional and functional structures of the human oral [125,126] and gut [127,128,129] microbiomes.

Diet influences the oral and gut microbiomes beginning at birth, with human milk oligosaccharides involved in the assembly and maturation of the microbiota in early childhood [130,131]. During weaning, the introduction of solid foods leads to an increase in microbial richness and complexity, typically associated with adulthood [132,133]. Once reached, the typical microbiome configuration of the adult is relatively stable and resilient over time; yet characterized by alternating periods of stationary dynamics and fluctuations due to host and transient environmental factors, and also diet [15,24,134]. The gut microbiota, specifically, remains overall stable during adulthood and begins to change in older age, with gradual losses in diversity and compositional rearrangements. This is attributable to a progressively poorer diet (especially in fiber), as well as to an increased sedentary lifestyle, immunosenescence, and inflammaging [135,136].

Several studies have compared populations with disparate lifestyles, such as Westerners vs. residents from rural Venezuela [66] and Papua New Guinea [57], and ancestral populations still following the Paleolithic-type diet as the Hadza hunter-gatherers [54]. Significant differences in gut microbiome composition were found to be correlated with subsistence practices and geography [137].

Taken together, understanding the effects of specific nutrients on the microbiome is necessary for evaluating the core human microbiome. Different core functions may be required for different diets, making nutrition a potentially important factor in determining the core human microbiome. Several studies identified means by which various microbial groups respond to different diets. Generally speaking, a fiber-rich plant-derived diet is associated with enriched microorganisms endowed with fibrolytic capabilities, such as *Prevotella* from the Bacteroidetes phylum, and Lachnospiraceae and Ruminococcaceae members from Firmicutes [63,66]. These fiber-related specific signatures appear as clearly associated with healthy dietary patterns such as those associated with vegan or vegetarian diets [138] or the Mediterranean diet [139,140]. Conversely, Western high-protein high-fat diets are typically associated with bile-tolerant and fat-loving microbes (e.g., *Bacteroides*, *Bilophila*, *Collinsella*, etc.), as well as bacteria capable of utilizing host mucus, which serves as a backup food source when dietary fiber is limited or unavailable [127,141,142]. Non-digestible dietary carbohydrates, otherwise known as microbiota-accessible carbohydrates [141], are fermented by the colonic microbiota, thus producing short-chain fatty acids (SCFAs) [143]. These bioactive small molecules are strongly associated with a multitude of beneficial effects for the human host [144,145,146]. On the other hand, the gut microbiota can metabolize protein substrates into branched-chain fatty acids and phenolic and indole metabolites, which are variously associated with poor health outcomes [146]. A paradigmatic example of a maladaptive diet–microbiota–host interaction is represented by trimethylamine (TMA), which is formed by the microbiota starting from choline and carnitine present in cheese, eggs, red meat, and seafood. Once absorbed from the gut, TMA circulates to the liver where it is oxidized by host enzymes into TMA-N-oxide, a major cardiovascular risk factor [147]. Consistently, lower levels of TMA-N have been found in vegans and vegetarians than in omnivore subjects [139]. The most recent studies performed by metagenomics and the use of the up-to-date genome reconstruction pipelines mentioned above indicate that the diet affects the gut microbiome up to the strain-level dimension. Indeed, diet-sensitive and diet-associated microbial taxa that are widely studied in the human gut microbiome, such as *Prevotella* and *Faecalibacterium,* have demonstrated great genome-level variability according to the type of diet or the level of industrialization of the studied population [42,148,149]. Genome-level analysis can also be useful for tracing the possible occurrence in the gut of microorganisms that can be acquired through the consumption of certain foods such as lactic acid bacteria from fermented foods [42,150].

To the best of our knowledge, nutrition by itself is not usually considered an aspect of core human microbiome studies, except for defining populations that are assumed to have separate core microbiomes. Different diets require different functions from the microbiome, many of which must be fulfilled by the core microbiome. Considering nutrition information in core microbiome analysis is currently challenging, because accurate dietary information of individuals is usually not available, and the effects of specific nutrients on specific microbial species are generally not known. Given the high complexity of defining the activity of a certain dietary component (as a substrate) in each individual, such knowledge is unlikely to become available in the coming years for most nutrients and the microbes that process them.

### 5.2. The Immune System and Its Interactions with the Microbiome

The human body and our microbiome have co-evolved towards mutualism and homeostasis [151]. This coexistence is fundamental for the fine-tuning of the immune system and requires an intimate relation with commensal microbes, especially during early childhood [152,153]. Therefore, it is not surprising that alterations in these close microbiome–immunity interactions are associated with increased susceptibility to pathogenic infections, overgrowth of commensals, and their potential spread throughout the body via impaired mucosal barriers [154,155]. These contribute to the onset and development of several disorders, including IBD [45], rheumatic arthritis [156], neurodegenerative diseases [157], and even tumors [158]. 

The crosstalk between the host’s immune system and the microbiome is extremely complex and involves microbial structural components, e.g., lipopolysaccharide, lipoteichoic acid, microbiome-derived metabolites such as SCFAs, indole derivatives acting as aryl hydrocarbon receptor ligands [146], host-derived molecules including non-specific antimicrobial peptides [159,160], mucosal immunoglobulins IgA [161], and miRNAs that act over bacterial transcripts regulating their growth [162]. 

The hosts’ local environments are sensed by commensal microorganisms to induce responses that enable their avoidance, escape, or tampering with the innate immune mechanism of defense [163,164]. Beyond the importance of adaptation to the environment, an array of microbial-derived molecules can promote commensal processes that are beneficial for both the host and the microbiome; and conversely, the host immune system recognizes the commensal antigen patterns and adapts to them. Microbial components are sensed by pattern-recognition receptors, including toll-like receptors and NOD-like receptors. These promote several mechanisms of tolerance, while preventing infections by harmful organisms [4,153]. 

SCFAs induce the production of anti-inflammatory cytokines, driving the local increase of Tregs [165,166]. In particular, butyrate acts as an inhibitor of histone deacetylase, epigenetically modulating immune system function, including Foxp3 gene expression [167]. Moreover, propionate has been shown to regulate allergic inflammation, bone marrow hematopoiesis, and dendritic cell function [168]. Several other microbiome-derived compounds, such as polyamines, can inhibit the macrophagic production of pro-inflammatory cytokines [169]. Microbial metabolic activity is sensed by the host immune system [170] and the information is converted into immune signaling pathways that can, in turn, stimulate anti-microbial activity or suppress the local immune response, to ultimately maintain the intimate cross-talk required for homeostasis. 

Given its significant interactions with the human microbiome, the immune system is likely involved in defining the core microbiome. As in the example of nutrition, the interactions between the microbiome and the immune system are not taken into account in core microbiome studies. Obtaining accurate data that is related to the immune system may become a more feasible task, and high-throughput methods for characterizing the immune response to microbial species have already been successfully implemented [171]. Such data would enable a better evaluation of the core microbiome in the context of the immune system.

### 5.3. The Less Explored Members of the Microbiome

Eukaryotes, eukaryotic viruses, and phages are all part of the human microbiome but currently, relatively little is known about their contribution to the core human microbiome, both as members and in shaping the core microbiome. 

#### 5.3.1. The Role of Eukaryotes in the Human Microbiome

Eukaryotes, especially lower-order ones such as protists, fungi, stramenopiles, and helminths, belong to the human gut ecosystem [172,173]. In the past, any protist or helminth species identified in humans was considered a parasite with an obvious harmful effect on its host. However, a number of human-associated lower eukaryotes are now known to play various roles in both health and disease [174]. For example, some stramenopile species (*Blastocystis* sp.), commonly considered parasites, have been found to be associated with healthy and diverse gut microbiomes [175]; and some helminths and protists are able to stimulate a beneficial immune response in individuals with allergies [176]. Despite their active role in shaping the gut microbiome, unicellular and multicellular eukaryotes are often neglected from microbiome studies. Some major limitations towards eukaryotes in microbiomes include: (i) a limited number of reference eukaryotic species with sequenced genomes, (ii) the common presentation of eukaryotic species at lower abundances than bacterial species and thus the likelihood that they are missed if the sequencing is not deep, (iii) the high diversity and poor taxonomic knowledge of many eukaryotes [177], especially in fungi and protists, for which accurate genus and species level are less determined [178], (iv) the need to elucidate the role of eukaryotes in maintaining eubiosis and homeostasis [179,180], and (v) the lack of standardized methodologies and sequencing designs [181]. Furthermore, eukaryotes are generally not identified with common bacterial 16S rDNA primers, and most universal 18S rDNA primers are limited in their ability to amplify a broad spectrum of eukaryotes. Nonetheless, specific primer combinations and standardization have proven useful sets for stool-based detection of gut eukaryotes [182]. In addition, approaches using RNA shotgun sequencing or “meta-ribosomalomics” of stool samples have successfully detected intestinal eukaryotes that have often been excluded from traditional routine diagnostic protocols [183,184].

Eukaryotes, such as the fungal genus *Candida* or *Rhodotorula*, are components of the core microbiome or contribute to its shaping. In Africa, intestinal helminths are common and are estimated to affect a large proportion of the population [185]. *Blastocystis* sp. were detected in 24% of the sampled population in the Czech Republic [186], suggesting that certain eukaryotes are also prevalent in Western countries. Current development of better bioinformatics algorithms, and databases with eukaryotic genomes, are expected to increase the feasibility of evaluating the contribution of eukaryotes to the core microbiome.

#### 5.3.2. The Host Eukaryotic Virome and the Gut Microbiome

More than 1000 eukaryotic virus species are known that can enter and live within the human body, forming the human eukaryotic virome [187,188]. Human viruses can be present in the gut as residential members, as transient passengers, as commensals, and as an integral part of the microbiome community. Interactions between the eukaryotic microbiome and the other members of the gut microbiome can be direct or indirect, and either cooperative or antagonistic. Multiple examples of these complex interactions are known.

The presence of eukaryotic enteric viruses can lead to changes in the bacterial gut microbiome, as in the example of rotavirus infections, which can induce changes within the composition of the *Bacteroides* genus [189]. More recently, SARS-CoV-2 attachment to the colonic ACE2 receptor in the presence of *Coprobacillus* was reported to lead to a reduction in the ‘good’ gut bacteria *Faecalibacterium*, *Roseburia*, *Bacteroides,* and *Lachnospiraceae;* and to increased pathogenic genera such as *Streptococcus*, *Rothia*, and *Veillonella* [190]. Furthermore, these shifts during and even after SARS-CoV-2 infections give rise to opportunistic bacteria such as *Corynebacterium* and *Ruthenibacterium* [191,192]. Eukaryotic viruses can also activate the host immune system that drives the overall composition of the gut microbiome, as in the example of influenza viruses that invade the respiratory tract and induce a systemic type 1 IFN-1 response. This leads to a reduction in anaerobes and improvement of *Enterobacteriaceae* in the gut microbiome [193]. 

In contrast to the above, the gut microbiome can affect the infection processes of various eukaryotic enteric viruses in the gastrointestinal tract [194] and can induce an immune response that controls the infection process of eukaryotic viruses [195]. The enteric microbiota enhances the production of mucus and the synthesis of potential antiviral compounds, such as lactic acid, acetic acid, reactive oxygen species, and defensins, which inhibit local viral replication [196]. In addition, small bacterial proteins and bacteriocins, e.g., surfactin, a cyclic lipopeptide produced by *Bacillus subtilis*, can destroy eukaryotic virus integrity. This effect has been described for several enveloped viruses, including Chikungunya, Crimean–Congo hemorrhagic fever, Dugbe, Ebola, Zika, and coronaviruses [197].

The core microbiome may be involved in defending the human host against eukaryotic viruses. The stability of the core microbiome depends on resisting these viruses and the direct and indirect ways in which they affect the microbiome. The eukaryotic virome cannot be studied through 16S rRNA gene surveys and is rarely addressed in metagenomics studies. Improving bioinformatics methods and reference databases should be a target for evaluating the roles and interactions of these viruses with the core microbiome.

#### 5.3.3. The Prokaryotic Virome (Phageome) and Its Relation to the Gut Microbiome

The bacterial virome (phageome) of the gut is composed of free phages (virus-like particles) and prophages and is one of the most complex and poorly understood consortia in the context of the gut microbiome. New scientific work indicates that the pan gut bacterial virome is composed of more than 50,000 candidate viral species [198], and up to 140,000 different phages [199]. The number of bacteria in the microbiome and the number of virus-like particles (up to 10^12^ per gram of feces) are in about equal proportion [200]. Some large bacterial phages can add more than 500 kbp of genetic information to their host’s genome [201].

Phages can be involved in the core microbiome in various ways. Phages that infect multiple species can be part of gene flow networks across phylogenetically distant bacterial species [202]. This can result in the distribution of core functions between multiple species and increase the stability of the core microbiome. Lysogenic phages can contribute new traits and physical/biochemical properties to their host [203]. As a result, certain core functions may be contributed by phages. Phages/prophages control bacterial cell death by indirectly gaining information of environmental factors such as community composition, nutrient availability, and local bacterial cell density via bacterial quorum sensing [202,204,205]. Phage-programmed bacterial cell death also leads to redistribution of nutrients [201] and, consequently, sometimes to remarkable shifts in the metabolome composition. To this end, phages can serve as both a threat to the core microbiome and as a means of eliminating transient members of the community. The exact roles of the gut phageome with respect to the core microbiome are poorly understood. Work carried out by researchers and data scientists towards developing models of core and healthy phageomes [206] may shed light on this.

## 6. Discussion

The core human microbiome has been the subject of multiple studies due to its potential significance in health and disease. Still, there is currently no consensus as to whether the core human microbiome should be defined at the level of taxa or functions, or some other level; the main forces that shape it, and the consequences of an impaired core microbiome. One major limitation in core microbiome research is the predominantly one-dimensional approach, which focuses on taxa and gene-based functions at the genomic level. Considering other data layers, both regarding the microbiome and the host, will likely help expose relations between these layers that are relevant to the roles and definitions of the core microbiome. Evidently, the core human microbiome should be discussed in the context of its environment. Such contexts include body parts, diet, geography, age, health state, and possibly other factors. Once the true nature of the core human microbiome is understood, accurate definitions and terminology can be developed that will help to uncover the relevance of the core human microbiome to biological, ecological, and evolutionary processes that occur in the human host and its microbiome.

Given the efforts invested and the amount of available data, the core human microbiome is unlikely to be defined by a fixed set of taxa, as no such consistent set has been found. A set of universal functions shared among humans may be identified; however, many of these functions are also shared by other organisms, including non-mammals. A set of functions likely exists that distinguishes our microbiome as “human”, but more research to characterize those functions is required. Missing annotations to a large proportion of known and newly discovered genes is an obstacle that may be overcome by homology-based clustering of all known genes and proteins, regardless of their annotation. Incorporating the hundreds of thousands of MAGs published in recent years into public reference databases is expected to contribute to the establishment of a comprehensive set of genes associated with the microbiome of the gut and other parts of the body. Finally, analyzing functions in the context of their genomes can reveal sets of redundant taxa that define a core microbiome. Programs such as HUMMAnN3 already provide this type of information as part of their output, and more comprehensive reference databases can provide a proper basis for such attempts.

The study of the core human microbiome is currently restricted mostly to NGS-based approaches that, together with proper bioinformatics methods, generate large amounts of data. Of the two most used approaches, amplicon surveys and metagenomics, the latter can provide the necessary species- and even strain-level taxonomic and functional resolution required. Long-read technologies have the potential to improve and simplify the metagenomics analysis process by enabling a more accurate genome recovery process for a larger portion of the community. We anticipate that the coming years will witness addressing shortfalls such as incomplete reference databases; failure to assess the abundance of eukaryotes, eukaryotic viruses, and phages; and the insufficient number of samples from non-Western populations. Other technical challenges such as direct sampling from different parts of the gut currently do not have easy technical solutions. Therefore, the availability of highly localized data may remain low in the near future, limiting our ability to accurately evaluate the core microbiome of the various niches in the gut. Collecting, processing, and analyzing meta-omics data at levels other than the genomic can be challenging. Metatranscriptomic data, for example, are dominated by ribosomal RNA, and mRNA is very unstable, both issues requiring technical solutions that may be challenging. In addition, analysis pipelines for such data still require improvement. As for metabolomics, proton nuclear magnetic resonance (1H NMR), one of the main analytical tools used in metabolomics studies, suffers from low sensitivity and may not detect low-abundance metabolites [207,208]. Besides NMR, the chromatographic approach (whether gas or liquid at high performance) has been effectively used coupled with quadrupole-TOF (time of flight) mass spectrometry [209] to cover a high number of metabolites from microbial samples. However, TOF-based detection conventionally requires complex sample preparation and chromatographic fractionation routines while providing only a reasonable resolution in discovery-based metabolomics. To overcome these limitations, novel high-resolution accurate-mass (HRAM) analytical platforms have been implemented in the field of complex microbial community metabolomics. For example, ultra-high performance liquid chromatography (UHPLC) and quadrupole-orbitrap mass spectrometer allowed the untargeted identification of novel potential metabolites [210,211]. The orbitrap mass analyzer—despite being an expensive instrument that requires complex data analysis skills—enhances the separation of unknown and known compounds enabling high-throughput workflows and constitutes to date the most advanced platform used in discovery-based proteomics and metabolomics, providing valuable tools in facing the unknowns in host-microbiome metabolomics [212]. 

Overall, we expect our knowledge of the transcriptomic, proteomic, and metabolomic levels to significantly improve in the coming years, though the rate of data generation is not expected to achieve that of amplicon and even metagenomics data. Method development for integrating multi-omics data is still required to generate multi-layer models of the core human microbiome.

Challenges in characterizing the core human microbiome may be due to our limited understanding of the exact interactions between the human microbiome and its environment, particularly diet and the immune system. As currently, no high-throughput technology provides the necessary information, accounting for these factors in the context of the core microbiome is difficult. The collection of more non-genomic data necessitates computational and statistical approaches to combine data layers and to identify complex patterns in the data. Machine learning is a promising direction that can help reveal such patterns that are difficult to identify by humans. Machine learning has been used to predict disease states, identify biomarkers, and more [213,214]. Interpretable machine learning algorithms such as decision trees and random forest can be used to identify features that are most relevant to groups of samples. For example, functions that are most relevant to the human microbiomes may be identified by comparing samples from humans against samples from mice, gorillas, and other animals. These and other algorithms may identify patterns that involve multiple features (e.g., functions) and are therefore more difficult to discover using standard statistical methods. Clustering algorithms may be used to define the core human microbiome in terms of groups of replaceable species by identifying co-occurring or mutually exclusive microbes. In addition, cloud computing and other high-throughput computational technologies make it feasible to analyze huge amounts of data, potentially even all the data stored in public databases [215]. This requires resolving issues related to the availability of metadata for the various samples. Still, domain expertise in nutrition, medicine, and immunology must be considered to interpret and evaluate the results. Therefore, collaborative efforts between experts in nutrition, immunology, human genetics, the microbiome, bioinformaticians, and machine learning can help design holistic studies that will uncover the patterns and forces that shape the core human microbiome.

## 7. Conclusions

The concept of the core human microbiome should be considered in the context of its environment, including body parts, diet, and geography. More data and better bioinformatics are expected to improve our abilities to assess the core human microbiome based on metagenomics, while the contribution of 16S rRNA gene surveys may remain limited. The analysis of previously less explored members of the microbiome will benefit from these trends and enable a more complete view of the core human microbiome. The genomic data should be integrated with other omics data; the technology to collect these data exists, but the bioinformatics and available public data are still lagging. Non-omics data related to diet and immunology are desired, but achieving such data is expected to remain challenging. Implementation of machine learning approaches on large amounts of data may reveal patterns that are otherwise difficult to identify. A collaborative effort of multidisciplinary teams will likely be needed to advance understanding of the core human microbiome. 

## Figures and Tables

**Table 1:** Approaches for defining the core human microbiome.

Approach	Pros	Cons	Examples
**Community composition**: the core microbiome is described in terms of shared taxa	Relatively simple to implement; can be applied to amplicon studies	Common taxa are usually identified at high taxonomic levels only	[17,28,29,30,31]
**Functional profile**: definition relies on a set of common functions	Captures the contribution of the core human microbiome to the host and the community	It is difficult to distinguish between human-specific and broad core functions	[9,12,13,26]
**Ecology:** include taxon abundance, interactions, co-occurrence, and other community-level patterns	Can capture complex patterns in community structure; may be more realistic than community composition alone	Less clear which patterns should be considered; no standard methods and programs are available	[19]
**Stability:** consider factors that maintain community stability and resilience	Stability is a critical characteristic of the core microbiome that is not captured through community composition alone	Definition is vague; there are no widely accepted methods for evaluating stability and resilience	[32]

**Table 2:** A summary of the challenges that affect our ability to characterize the core human microbiome.

**Data (** Section 3 **)**	
Technology	Hundreds of thousands of human-associated 16S and metagenomics samples. Only thousands of other meta-omics samples
Population	Mostly Westerners. Agricultural and traditional populations are significantly underrepresented
Body part	Mostly fecal samples. The gut environment consists of multiple niches, each may have its own core microbiome
**Methods (** Section 4 **)**	
16S rRNA surveys	Hundreds of thousands of public 16S samples are available. The method is inexpensive with established lab procedures and bioinformatics pipelines. The data provides only low taxonomic-resolution community composition and no functional information
Metagenomics bioinformatics	Tens of thousands of public human-associated metagenomes available. The data can provide strain-level and functional information. Bioinformatics analysis is complex, reference databases still lack a significant portion of human-associated microbial species.
Difficult to mine public databases	Available metadata is typically partial, performing meta-analysis requires significant manual effort
**Beyond prokaryotic omics (** Section 5 **)**	
Diet	Both diet and the immune system shape the gut microbiome and are related to the functions it provides. Small amounts of data are available for both, with no high-throughput methods available for data collection
Microbiome–immune system interactions	
Non-prokaryotic members of the microbiome	Eukaryotes and viruses may be either part of the core human microbiome or related to the functions it provides. Both groups are invisible in 16S studies, current metagenomics bioinformatics mostly ignores them

## Data Availability

The data presented in this study are available in the Supplementary text.

## Supplementary Materials

The following supporting information can be downloaded at: https://www.mdpi.com/article/10.3390/nu14142872/s1, Figure S1: Functional profiling of eight cohorts; Table S1: Summary of information of the data that was used for the community and function analyses; Table S2: Statistics of samples related to the human microbiome; Table S3: fraction of samples in each cohort in which the species was identified; Table S4: UniRef90 clusters that are present at ≥90% of the samples of at least one cohort; Table S5: all MetaCyc pathways detected in at least one sample. References [9,28,37,38,39,40,41,216] are cited in the supplementary materials.
