# Supplementary material for: The Core Human Microbiome: Does It Exist and How Can We Find It? A Critical Review of the Concept
Source: Nutrients. 2022 Jul 13;14(14):2872. doi: 10.3390/nu14142872 (PMC9323970; doi:10.3390/nu14142872)
Supplement: Supplementary file 1 [file nutrients-14-02872-s001.zip › Core-microbiome.manuscript.supp.pdf]

Supplementary Information

The core human microbiome: does it exist and how can we find it? A critical review of the concept

Itai Sharon, Narciso Martín Quijada, Edoardo Pasolli, Marco Fabbrini, Francesco Vitali, Valeria Agamennone, Andreas Dötsch, Evelyne Selberherr, José Horacio Grau, Martin Meixner, Karsten Liere, Danilo Ercolini, Carlotta de Filippo, Giovanna Caderni, Patrizia Brigidi, Silvia Turroni

Methods

Sample selection and preparation. We selected samples from eight microbiome projects, which represent healthy westerners from the US (HMP 1, HMP 2, HMP 3) (1,2) and Denmark (MetaHit), IBD patients from Spain (MetaHit) (3), hunter-gatherers and traditional Bantu agriculturalists (4), and, as controls, gorillas (4), mice (5) and chicken (6). From each project, samples with at least 20,000,000 (20M) paired reads of length at least 50bp were considered. For these samples, the first 20M forward reads were used for the analyses. Maximum read size was set to 101bp by removing read ends if necessary. Information about the datasets used is summarized in Table S1.

Community profiling. Community composition was evaluated for each sample as follows. First, reads from the samples were aligned against CHOCOPhlAn v30 database using bowtie2 v2.3.5.1 (7) using the flags --sam-no-hd --sam-no-sq --no-unal --very-sensitive. Next, the mapped reads were used as input for MetaPhlAn v3.0 (8) with default parameters.

Functional profiling. We used humann v3.0.0 (8) with default parameters to generate gene- and pathway-level functional profiles of the samples. Number of reads per function was normalized using the humann_renorm_table script that is available as part of the bioBakery package. For our analysis that is summarized in **Fig. S1**, we considered pathways that appear in the samples, regardless of their species of origin.

Mining the ENA for samples related to the human microbiome: we identified 23 taxonomic terms that are related to the human microbiome in NCBI's taxonomy database. For these terms, we collected information for all the runs associated with each one using ENA APIs. The number of samples for each set of runs was calculated, as well as the number of reads and basepairs. Information is summarized in **Table S2**. To search for RNA-Seq samples, we search for "RNA-Seq" in the titles of the different studies.

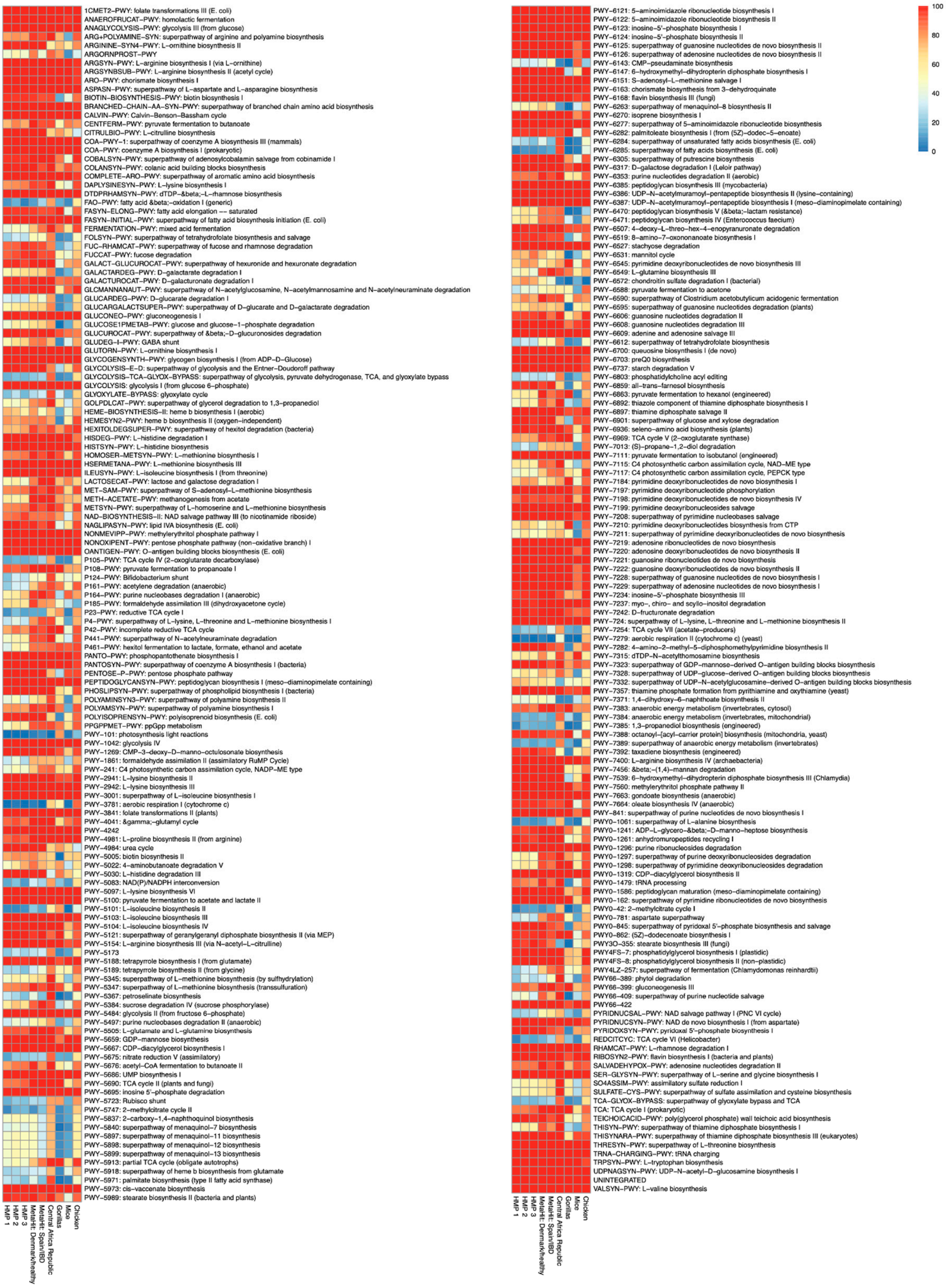

Figure S1: Functional profiling of eight cohorts: HMP phases 1 (n=138), 2 (n=91) and 3 (n=42), healthy individuals from Denmark (n=64), IBD patients from Spain (n=16), hunter-gatherers and traditional agriculturalists (n=19), gorillas (n=15), mice (n=141) and chicken (n=121). Colors indicate the fraction of samples for a project in which a pathway was detected.

List of samples used from each dataset

HMP 1:
SAMN00032498, SAMN00032521, SAMN00032571, SAMN00032676, SAMN00032708, SAMN00032739, SAMN00032842, SAMN00032966, SAMN00033023, SAMN00033491, SAMN00034120, SAMN00034376, SAMN00034433, SAMN00034694, SAMN00034739, SAMN00034905, SAMN00035018, SAMN00035169, SAMN00035453, SAMN00035505, SAMN00035531, SAMN00035677, SAMN00035831, SAMN00036197, SAMN00036283, SAMN00036351, SAMN00036408, SAMN00036435, SAMN00036482, SAMN00036587, SAMN00036649, SAMN00036796, SAMN00036881, SAMN00037000, SAMN00037072, SAMN00037108, SAMN00037178, SAMN00037236, SAMN00037274, SAMN00037313, SAMN00037485, SAMN00037553, SAMN00037713, SAMN00037735, SAMN00037803, SAMN00037971, SAMN00038172, SAMN00038207, SAMN00038321, SAMN00038409, SAMN00038465, SAMN00038525, SAMN00038651, SAMN00038739, SAMN00038919, SAMN00039039, SAMN00039351, SAMN00039531, SAMN00039569, SAMN00039645, SAMN00039793, SAMN00039874, SAMN00040035, SAMN00040202, SAMN00040248, SAMN00040286, SAMN00040379, SAMN00040485, SAMN00040599, SAMN00040615, SAMN00040800, SAMN00040819, SAMN00040903, SAMN00041005, SAMN00041128, SAMN00041186, SAMN00041451, SAMN00041546, SAMN00042087, SAMN00042702, SAMN00043166, SAMN00043289, SAMN00043355, SAMN00043827, SAMN00043931, SAMN00044394, SAMN00044564, SAMN00044744, SAMN00044801, SAMN00045047, SAMN00045132, SAMN00045189, SAMN00045227, SAMN00045293, SAMN00045350, SAMN00045483, SAMN00045843, SAMN00062293, SAMN00062637, SAMN00063010, SAMN00063420, SAMN00063710, SAMN00065013, SAMN00065722, SAMN00067023, SAMN00067053, SAMN00068173, SAMN00068879, SAMN00069173, SAMN00069721, SAMN00069909, SAMN00069968, SAMN00070004, SAMN00070308, SAMN00070431, SAMN00070761, SAMN00070934, SAMN00071040, SAMN00072036, SAMN00072706, SAMN00073223, SAMN00073344, SAMN00073407, SAMN00074599, SAMN00074965, SAMN00076268, SAMN00076528, SAMN00077487, SAMN00077726, SAMN00078779, SAMN00081451, SAMN00083009, SAMN00083300, SAMN00083581, SAMN00084528, SAMN00085565, SAMN00087897, SAMN00088343

HMP 2:
SAMN00031604, SAMN00034067, SAMN00034187, SAMN00034316, SAMN00034856, SAMN00034857, SAMN00037655, SAMN00037656, SAMN00039134, SAMN00041840, SAMN00042371, SAMN00042437, SAMN00045881, SAMN00063676, SAMN00065537, SAMN00065748, SAMN00069411, SAMN00069905, SAMN00070035, SAMN00073365, SAMN00073582, SAMN00073658, SAMN00074361, SAMN00075542, SAMN00076282, SAMN00078079, SAMN00082513, SAMN00082542, SAMN00083997, SAMN00084837, SAMN00085131, SAMN00085245, SAMN00085508, SAMN00087096, SAMN00087253, SAMN00087361, SAMN00087461, SAMN00087502, SAMN00087669, SAMN00087719, SAMN00087920, SAMN00088016, SAMN00088409, SAMN00088586, SAMN00088832, SAMN00094410, SAMN00095035, SAMN00095092, SAMN00095165, SAMN00095238, SAMN00095348, SAMN00096542, SAMN00097897, SAMN00097954, SAMN00099612, SAMN00099822, SAMN00099936, SAMN00100025, SAMN00100110, SAMN00139709, SAMN00139730, SAMN00139862, SAMN00141720, SAMN00141722, SAMN00141816,

SAMN00141929, SAMN00142107, SAMN00142287, SAMN00142398, SAMN00142559, SAMN00142634, SAMN00142815, SAMN00142997, SAMN00143093, SAMN00143208, SAMN00143579, SAMN00143723, SAMN00143754, SAMN00144714, SAMN00145981, SAMN00146029, SAMN00146030, SAMN00146239, SAMN00146356, SAMN00146563, SAMN00146869, SAMN00146983, SAMN00147136, SAMN00147413, SAMN00147641, SAMN00147938

HMP 3:
SAMN03351444, SAMN03351447, SAMN03351451, SAMN03351452, SAMN03351453, SAMN03351461, SAMN03351466, SAMN03351468, SAMN03351479, SAMN03351480, SAMN03351481, SAMN03351484, SAMN03351485, SAMN03351489, SAMN03351491, SAMN03351493, SAMN03351494, SAMN03351496, SAMN03351497, SAMN03351506, SAMN03351513, SAMN03351532, SAMN03351543, SAMN03351544, SAMN03351555, SAMN03351563, SAMN03351571, SAMN03351572, SAMN03351584, SAMN03351595, SAMN03351599, SAMN03351602, SAMN03351627, SAMN03351633, SAMN03351654, SAMN03351660, SAMN03351666, SAMN03351672, SAMN03351677, SAMN03351703, SAMN03351713, SAMN03351718

MetaHit/Denmark, healthy:
SAMEA728578, SAMEA728582, SAMEA728586, SAMEA728602, SAMEA728611, SAMEA728614, SAMEA728617, SAMEA728622, SAMEA728627, SAMEA728630, SAMEA728635, SAMEA728643, SAMEA728655, SAMEA728658, SAMEA728661, SAMEA728667, SAMEA728670, SAMEA728672, SAMEA728674, SAMEA728676, SAMEA728680, SAMEA728690, SAMEA728697, SAMEA728700, SAMEA728723, SAMEA728726, SAMEA728728, SAMEA728734, SAMEA728737, SAMEA728740, SAMEA728749, SAMEA728752, SAMEA728760, SAMEA728763, SAMEA728772, SAMEA728774, SAMEA728780, SAMEA728785, SAMEA728794, SAMEA728799, SAMEA728802, SAMEA728808, SAMEA728812, SAMEA728814, SAMEA728826, SAMEA728829, SAMEA728835, SAMEA728848, SAMEA728851, SAMEA728854, SAMEA728856, SAMEA728859, SAMEA728865, SAMEA728887, SAMEA728890, SAMEA728899, SAMEA728902, SAMEA728908, SAMEA728916, SAMEA728918, SAMEA728925, SAMEA728929, SAMEA728933, SAMEA728946

MetaHit/Spain, IBD:
SAMEA728570, SAMEA728574, SAMEA728649, SAMEA728652, SAMEA728665, SAMEA728709, SAMEA728717, SAMEA728720, SAMEA728758, SAMEA728769, SAMEA728788, SAMEA728791, SAMEA728797, SAMEA728805, SAMEA728845, SAMEA728873

Central Africa Republic:
SAMN15047040, SAMN15047043, SAMN15047046, SAMN15047047, SAMN15047048, SAMN15047051, SAMN15047053, SAMN15047054, SAMN15047055, SAMN15047056, SAMN15047057, SAMN15047059, SAMN15047060, SAMN15047061, SAMN15047062, SAMN15047063, SAMN15047065, SAMN15047066, SAMN15047067

Gorillas:
SAMN15047018, SAMN15047019, SAMN15047022, SAMN15047023, SAMN15047025, SAMN15047027, SAMN15047029, SAMN15047030, SAMN15047031, SAMN15047032, SAMN15047034, SAMN15047035, SAMN15047036, SAMN15047038, SAMN15047039

Mice:
SAMEA3134357, SAMEA3134358, SAMEA3134359, SAMEA3134360, SAMEA3134361, SAMEA3134362, SAMEA3134363, SAMEA3134366, SAMEA3134368, SAMEA3134369, SAMEA3134370, SAMEA3134371, SAMEA3134372, SAMEA3134374, SAMEA3134375, SAMEA3134376, SAMEA3134377, SAMEA3134378, SAMEA3134379, SAMEA3134380, SAMEA3134381, SAMEA3134382, SAMEA3134384, SAMEA3134386, SAMEA3134387, SAMEA3134389, SAMEA3134390, SAMEA3134391, SAMEA3134392, SAMEA3134397, SAMEA3134399, SAMEA3134400, SAMEA3134401, SAMEA3134402, SAMEA3134404, SAMEA3134405, SAMEA3134406, SAMEA3134407, SAMEA3134408, SAMEA3134409, SAMEA3134410, SAMEA3134411, SAMEA3134412, SAMEA3134413, SAMEA3134414, SAMEA3134415, SAMEA3134416, SAMEA3134417, SAMEA3134418, SAMEA3134419, SAMEA3134420, SAMEA3134421, SAMEA3134422, SAMEA3134424, SAMEA3134425, SAMEA3134426, SAMEA3134427, SAMEA3134428, SAMEA3134429, SAMEA3134430, SAMEA3134431, SAMEA3134432, SAMEA3134433, SAMEA3134434, SAMEA3134435, SAMEA3134436, SAMEA3134437, SAMEA3134438, SAMEA3134439, SAMEA3134440, SAMEA3134441, SAMEA3134442, SAMEA3134443, SAMEA3134444, SAMEA3134445, SAMEA3134446, SAMEA3134447, SAMEA3134448, SAMEA3134449, SAMEA3134450, SAMEA3134451, SAMEA3134452, SAMEA3134453, SAMEA3134454, SAMEA3134457, SAMEA3134459, SAMEA3134479, SAMEA3134480, SAMEA3134481, SAMEA3134482, SAMEA3134483, SAMEA3134484, SAMEA3134485, SAMEA3134486, SAMEA3134487, SAMEA3134488, SAMEA3134489, SAMEA3134490, SAMEA3134491, SAMEA3134492, SAMEA3134493, SAMEA3134494, SAMEA3134495, SAMEA3134496, SAMEA3134497, SAMEA3134498, SAMEA3134499, SAMEA3134500, SAMEA3134501, SAMEA3134502, SAMEA3134503, SAMEA3134504, SAMEA3134507, SAMEA3134509, SAMEA3134510, SAMEA3134511, SAMEA3134512, SAMEA3134514, SAMEA3134515, SAMEA3134518, SAMEA3134519, SAMEA3134520, SAMEA3134521, SAMEA3134522, SAMEA3134523, SAMEA3134524, SAMEA3134525, SAMEA3134526, SAMEA3134527, SAMEA3134528, SAMEA3134529, SAMEA3134530, SAMEA3134531, SAMEA3134532, SAMEA3134533, SAMEA3134534, SAMEA3134535, SAMEA3134536, SAMEA3134537, SAMEA3134538, SAMEA3134539

Chicken:
SAMN07672796, SAMN07672797, SAMN07672798, SAMN07672799, SAMN07672800, SAMN07672802, SAMN07672803, SAMN07672804, SAMN07672805, SAMN07672806, SAMN07672807, SAMN07672808, SAMN07672809, SAMN07672810, SAMN07672811, SAMN07672812, SAMN07672813, SAMN07672814, SAMN07672816, SAMN07672818, SAMN07672819, SAMN07672820, SAMN07672821, SAMN07672822, SAMN07672823, SAMN07672824, SAMN07672825, SAMN07672826, SAMN07672827, SAMN07672828, SAMN07672829, SAMN07672830, SAMN07672831, SAMN07672832, SAMN07672833, SAMN07672834, SAMN07672835, SAMN07672836, SAMN07672837, SAMN07672838, SAMN07672839, SAMN07672840, SAMN07672841, SAMN07672842, SAMN07672844, SAMN07672845, SAMN07672846, SAMN07672847, SAMN07672848, SAMN07672849, SAMN07672850, SAMN07672851, SAMN07672852, SAMN07672853, SAMN07672854, SAMN07672855, SAMN07672856, SAMN07672857, SAMN07672858, SAMN07672860, SAMN07672861, SAMN07672862, SAMN07672863, SAMN07672864, SAMN07672865,

SAMN07672866, SAMN07672867, SAMN07672868, SAMN07672869, SAMN07672870, SAMN07672871, SAMN07672872, SAMN07672873, SAMN07672874, SAMN07672875, SAMN07672876, SAMN07672877, SAMN07672878, SAMN07672879, SAMN07672880, SAMN07672881, SAMN07672883, SAMN07672884, SAMN07672885, SAMN07672886, SAMN07672887, SAMN07672888, SAMN07672889, SAMN07672890, SAMN07672891, SAMN07672892, SAMN07672893, SAMN07672894, SAMN07672895, SAMN07672896, SAMN07672897, SAMN07672898, SAMN07672899, SAMN07672901, SAMN07672902, SAMN07672903, SAMN07672904, SAMN07672905, SAMN07672906, SAMN07672907, SAMN07672908, SAMN07672909, SAMN07672910, SAMN07672911, SAMN07672912, SAMN07672913, SAMN07672914, SAMN07672915, SAMN07672917, SAMN07672918, SAMN07672919, SAMN07672920, SAMN07672921, SAMN07672922, SAMN07672924, SAMN07672925

References

1. Turnbaugh PJ, Ley RE, Hamady M, Fraser-Liggett CM, Knight R, Gordon JI. The human microbiome project. Nature. 2007 Oct 18;449(7164):804–10.
2. Lloyd-Price J, Mahurkar A, Rahnavard G, Crabtree J, Orvis J, Hall AB, et al. Strains, functions and dynamics in the expanded Human Microbiome Project. Nature. 2017 Oct 5;550(7674):61–6.
3. Qin J, Li R, Raes J, Arumugam M, Burgdorf KS, Manichanh C, et al. A human gut microbial gene catalogue established by metagenomic sequencing. Nature. 2010 Mar 4;464(7285):59–65.
4. Sharma AK, Petrzelkova K, Pafco B, Jost Robinson CA, Fuh T, Wilson BA, et al. Traditional human populations and nonhuman primates show parallel gut microbiome adaptations to analogous ecological conditions. mSystems. 2020 Dec 22;5(6).
5. Xiao L, Feng Q, Liang S, Sonne SB, Xia Z, Qiu X, et al. A catalog of the mouse gut metagenome. Nat Biotechnol. 2015 Oct;33(10):1103–8.
6. Feng Y, Wang Y, Zhu B, Gao GF, Guo Y, Hu Y. Metagenome-assembled genomes and gene catalog from the chicken gut microbiome aid in deciphering antibiotic resistomes. Commun Biol. 2021 Nov 18;4(1):1305.
7. Langmead B, Salzberg SL. Fast gapped-read alignment with Bowtie 2. Nat Methods. 2012 Mar 4;9(4):357–9.
8. Beghini F, McIver LJ, Blanco-Míguez A, Dubois L, Asnicar F, Maharjan S, et al. Integrating taxonomic, functional, and strain-level profiling of diverse microbial communities with bioBakery 3. eLife. 2021 May 4;10.
